# Relaxed Phylogenetics and Dating with Confidence

**DOI:** 10.1371/journal.pbio.0040088

**Published:** 2006-03-14

**Authors:** Alexei J Drummond, Simon Y. W Ho, Matthew J Phillips, Andrew Rambaut

**Affiliations:** **1**Department of Zoology, University of Oxford, Oxford, United Kingdom; Massey UniversityNew Zealand

## Abstract

In phylogenetics, the unrooted model of phylogeny and the strict molecular clock model are two extremes of a continuum. Despite their dominance in phylogenetic inference, it is evident that both are biologically unrealistic and that the real evolutionary process lies between these two extremes. Fortunately, intermediate models employing relaxed molecular clocks have been described. These models open the gate to a new field of “relaxed phylogenetics.” Here we introduce a new approach to performing relaxed phylogenetic analysis. We describe how it can be used to estimate phylogenies and divergence times in the face of uncertainty in evolutionary rates and calibration times. Our approach also provides a means for measuring the clocklikeness of datasets and comparing this measure between different genes and phylogenies. We find no significant rate autocorrelation among branches in three large datasets, suggesting that autocorrelated models are not necessarily suitable for these data. In addition, we place these datasets on the continuum of clocklikeness between a strict molecular clock and the alternative unrooted extreme. Finally, we present analyses of 102 bacterial, 106 yeast, 61 plant, 99 metazoan, and 500 primate alignments. From these we conclude that our method is phylogenetically more accurate and precise than the traditional unrooted model while adding the ability to infer a timescale to evolution.

## Abstract 2

This new method can simultaneously infer phylogeny and estimate the molecular clock. The authors run their method on several large alignments to show its phylogenetic accuracy and ability to infer a timescale to evolution.

## Introduction

From obscure beginnings, phylogenetics has become an essential tool for understanding molecular sequence variation. In the past decade, huge progress has been made in developing methods for inferring phylogenies and estimating divergence dates. This development has been characterized by increases, both in the complexity of the models used to describe molecular sequence evolution, and in the sophistication of the methods for analyzing these new models. Nevertheless, a well-known problem that has persistently troubled phylogenetic inference is that of substitution rate variation among lineages. In order to infer divergence dates, it is convenient to assume a constant rate of evolution throughout the tree [ 1, 2]. This practice has been regularly challenged by results from datasets showing considerable departures from clocklike evolution [ 3– 5], and rate variation among lineages can seriously mislead not only divergence date estimation [ 6] but also phylogenetic inference (e.g., [ 7, 8]).

Such problems with the molecular clock hypothesis have resulted in it being abandoned almost entirely for phylogenetic inference in favor of a model that assumes that every branch has an independent rate of molecular evolution. Under such an assumption, it is possible to infer phylogenies (e.g., [ 9, 10]), but not to estimate molecular rates or divergence times, because the individual contributions of rate and time to molecular evolution cannot be separated. If the rate and time along each branch can only be estimated as their product, then the position of the root of the tree cannot be estimated without additional assumptions such as an outgroup or a non-reversible substitution process. This unrooted alternative to the molecular clock was first suggested by Felsenstein [ 10] and has formed the basis of all modern phylogenetic inference and is implemented in all major phylogenetic packages (e.g., PHYLIP [ 11], PAUP* [ 12], and MrBayes [ 9]).

Recently, it has been realized that less drastic alternatives to the unrooted model of phylogeny may exist. Instead of dispensing with the molecular clock entirely, attempts have been made to relax the molecular clock assumption by allowing the rate to vary across the tree [ 13– 15]. For example, local molecular clock models estimate a separate molecular rate for each user-circumscribed group of branches in the tree [ 6, 13, 16]. However, assigning branches to different groups can be a difficult exercise if the number of sequences is large or if there is considerable uncertainty about the phylogenetic relationships among the taxa. Essentially, such models are only useful in cases in which there is a strong prior hypothesis that the rate of specific taxa will differ from the rest of the tree [ 6].

Bayesian relaxed-clock methods, including those published by Thorne et al. [ 15] and Aris-Brosou and Yang [ 17], present an enticing alternative to local clock models. These model the molecular rate among lineages as varying in an autocorrelated manner, with the rate in each branch being drawn (a priori) from a parametric distribution whose mean is a function of the rate on the parent branch. For example, a lognormal distribution can be employed with the variance scaled relative to the length of the branch in units of time, implying that the evolutionary rate changes continuously along the branch. Alternatively, the use of an exponential distribution would imply that changes occurred at the nodes, with the size of the change being independent of the branch length.

Autocorrelation of rates from ancestral to descendant lineages will occur whenever the largest component of rate variation is due to inherited factors, whether these are life-history traits or biochemical mechanisms. As one looks over smaller and smaller timescales, the differences in such inherited factors become smaller relative to the variance caused by stochastic and uninherited factors (such as environmental or chance events). An alternative way of considering this is that the autocorrelation is so strong that very little of the variation in rate can be attributed to inherited factors. At the other extreme, over very long timescales, we might expect so much variation in the inherited determinants of rate that the autocorrelation from lineage to lineage begins to break down, especially with sparse taxon sampling. However, it is difficult to predict where the boundaries between these effects are and thus to specify what the degree of autocorrelation will be.

Relaxed-clock models present a potentially useful method for removing the assumption of a strict molecular clock, but a major shortcoming of the methods that have been proposed thus far is that they require the user to specify the tree topology. This is a problem because in many cases, important parts of the tree may be uncertain or unresolved, resulting in a number of plausible tree topologies. Furthermore, a molecular clock may have been assumed when estimating the input tree (for example to find a root), but rate variation among lineages can adversely affect phylogenetic inference (e.g., [ 7, 8]). In some settings, the tree topology may actually be a nuisance parameter and some other aspect of the model (such as the variance in evolutionary rate, the effective population size, or the age of the most recent common ancestor) is the object of interest. Lastly, the assumption of a relaxed clock will alter the posterior probabilities of alternative tree topologies, so that the best tree under a relaxed-clock model may differ from the best tree under an unrooted or strict molecular clock model. For these reasons, a “relaxed phylogenetics” approach, in which the phylogeny and the divergence dates are co-estimated under a relaxed molecular clock, is preferred [ 18].

Here we present a Bayesian Markov chain Monte Carlo (MCMC) [ 19, 20] method for performing relaxed phylogenetics that is able to co-estimate phylogeny and divergence times under a new class of relaxed-clock models. Its utility is demonstrated through simulation and on 871 real datasets. When absolute rates and divergence dates are estimated, we use probabilistic calibration priors, rather than point calibrations, since these more appropriately incorporate calibration uncertainties. We have implemented this method in the application BEAST [ 21] in which they can be used in conjunction with a wide range of other evolutionary models.

## Results

### Simulations

**Figure 1:**
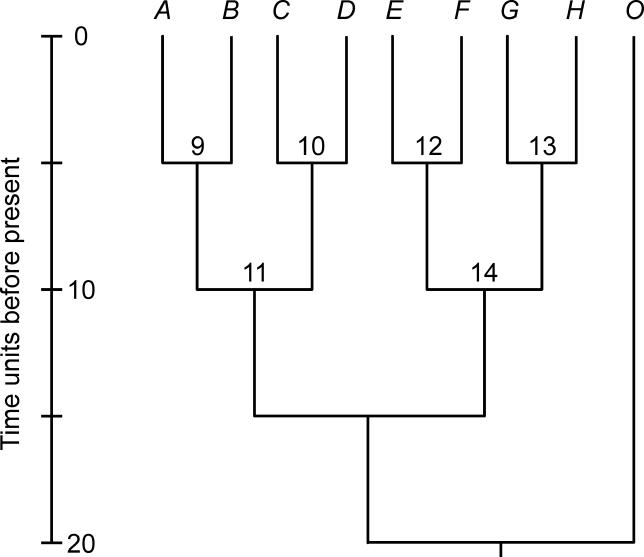
The Rooted Binary Tree Used for Simulating Sequence Evolution The timescale is drawn in arbitrary time units. Apart from the branch leading to the outgroup, sequence O, all branches are five time units in length.

We generated alignments of nine nucleotide sequences, each 1,000 nucleotides in length, on the rooted tree in Figure 1. The outgroup sequence, O, is only used for rooting; otherwise, the tree is symmetric. Simulations were performed using the program RateEvolver v1.0 [ 22], which can simulate nucleotide substitution under different rate conditions, including constant (molecular clock), autocorrelated, and uncorrelated rates.

Fifty sequence alignments were generated under each of five sets of rate variation models: (1) Rates were fixed at 0.01 average substitutions per site per time unit throughout the tree (i.e., rates conformed to a molecular clock) (CLOC); (2) rates were lognormally autocorrelated among branches, with an ancestral rate of 0.01 average substitutions per site per time unit and a variance parameter ( *S* ^2^) of 0.1, so that *S* ^2^ *t = 0.5* (ACLN); (3) rates were exponentially autocorrelated among branches, with an ancestral rate of 0.01 average substitutions per site (ACED); (4) rates were uncorrelated, with the rate in each branch independently drawn from a lognormal distribution with mean 0.01 and variance parameter of 0.5 (UCLN); and (5) rates were uncorrelated, with the rate in each branch independently drawn from an exponential distribution with mean (and therefore standard deviation) of 0.01 (UCED).

A normally distributed calibration prior with mean 20.0 and standard deviation 1.0 was specified for the age of the root of the tree, and the tree topology was fixed. Each alignment was analyzed using BEAST [ 21] with 5,000,000 steps, following a discarded burn-in of 500,000 steps. In each analysis, convergence of the chain to the stationary distribution was confirmed by inspection of the MCMC samples using the program Tracer 1.2 [ 23]. This application analyses posterior samples of continuous parameters from Bayesian MCMCs to allow visual inspection of the chain behavior, estimating of the effective sample size of parameters and the plotting of marginal posterior densities. The effective sample size is the number of independent samples that would be the equivalent to the autocorrelated samples produced by the MCMC. This provides a measure of whether the chain has been run for an adequate length (for example, if the effective sample sizes of all continuous parameters are greater than 200).

In four of the five cases, the uncorrelated relaxed-clock approach to estimating rates performed well ( Table 1). In all cases, the rate estimates made under the UCED had the largest 95% highest posterior densities (HPDs). This can be most clearly seen in the rates estimated from sequences generated under a molecular clock, with the average 95% HPD size under the UCED model exceeding that under the UCLN model by an order of magnitude.

**Table 1:**
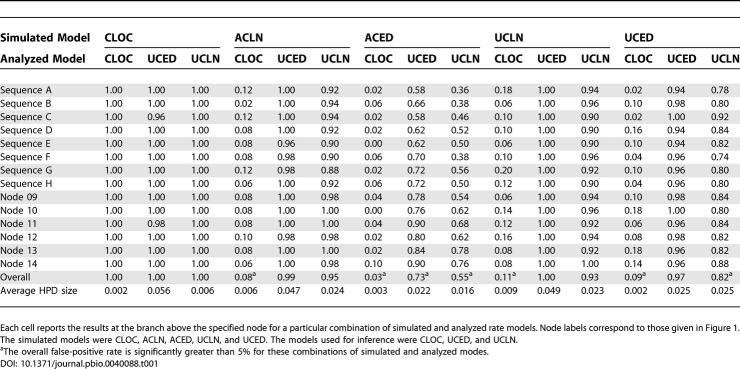
The Proportion of Datasets (50 Simulations for Each of Five Models) for Which the True Rate Was within the 95% HPD Limits at the Given Branch

When the sequences were simulated under a molecular clock, the 95% HPD interval of the posterior rate estimate almost always contained the true rate under all three analysis models ( Table 1). For sequences simulated under any of the other models, CLOC did extremely poorly, with the true rates included in the 95% HPDs between only 3% and 11% of the time. Clock estimates of rates from data generated under exponential rate models (ACED and UCED) were poorer than those from data generated under lognormal rate models (ACLN and UCLN); this was expected, since the variance of the exponential distribution is larger than those of the lognormal distributions in our simulations.

For the data generated under lognormal models (ACLN and UCLN), both of the uncorrelated models (UCED and UCLN) performed well with respect to coverage, with the 95% HPD containing the true rate between 93% and 100% of the time for individual branches. However, for the UCED model this was at the expense of power, with the average size of the HPDs being twice as large as those for the UCLN model.

For data generated under UCED, the UCED model performed better than UCLN with both models giving the same average size of HPDs, but with the latter model including the true rates in the HPDs slightly less often (82%). Neither model performed as well when the data were generated under an ACED model, with the true rate in the 95% HPD between 36% and 90% of the time.

The accurate estimation of molecular rates is important because it has a direct impact on the estimation of branch lengths, which can in turn affect the inferred tree topology. Collectively, the results provide a strong recommendation against assuming a molecular clock when analyzing data that have not evolved under clocklike conditions, but the uncorrelated relaxed-clock models also perform well when the data are clocklike. The results favor the use of the UCLN model in that it has an accuracy comparable to the UCED model, but it results in considerably smaller HPDs. In particular, because the UCLN model has the variance of the lognormal distribution as a parameter, it can better accommodate data that are close to being clocklike. This is not contradicting the findings of a previous simulation-based study [ 22], which suggested that the autocorrelated exponential model outperformed the lognormal model in rate estimation, because the uncorrelated models presented here are fundamentally different from autocorrelated models. Moreover, the previous simulation study considered only the accuracy of the estimates, and not their precision.

### Dengue Virus Type 4 and Human Influenza A Virus

We selected two virus datasets that were matched in the number of sequences ( *n* = 69) and the time span over which the data had been sampled (17 y). The first dataset was a previously published sequence alignment of the *E* gene of dengue-4 virus (1,485 base pairs [bp]) from Puerto Rico [ 24]. The second dataset was an alignment of hemagglutinin sequences from human influenza A virus selected to have a similar time frame (1981–1998; see Protocol S1 for details). In both of these datasets, each sequence in the alignment represents a consensus of the viral population within a single infected human host at the time of sampling. Therefore, both genealogies represent the ancestral relationships between the virus populations in a sample of 69 infected people spanning a 17-y period. These two viral datasets, particularly influenza A virus, are expected to exhibit the effects of natural selection, given the nature of their life histories [ 24– 26].

Both datasets were analyzed under the strict molecular clock and the UCLN and UCED models. For all analyses the HKY (Hasegawa-Kishino-Yano) model of nucleotide substitution [ 27] was used with gamma-distributed rate heterogeneity among sites [ 28]. Calibration information for the rate of evolution stems from the fact that each sequence has a date of sampling associated with it [ 29]. A constant-population coalescent prior was assumed [ 30]. For each combination of data and model, two independent MCMC analyses were each run for 10,000,000 steps, resulting in acceptable mixing as determined by Tracer 1.2 [ 23]. Given adequate sampling, these two runs were combined to obtain an estimate of the posterior distribution. The resulting estimates for the overall rate of evolution and coefficients of variation from the six analyses are presented in Table 2.

**Table 2:**
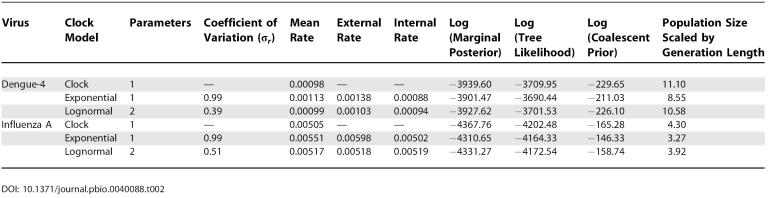
Rate and Variance Estimates for Two Viral Datasets (Human Influenza A Virus and Dengue Type-4 Virus)

**Figure 2:**
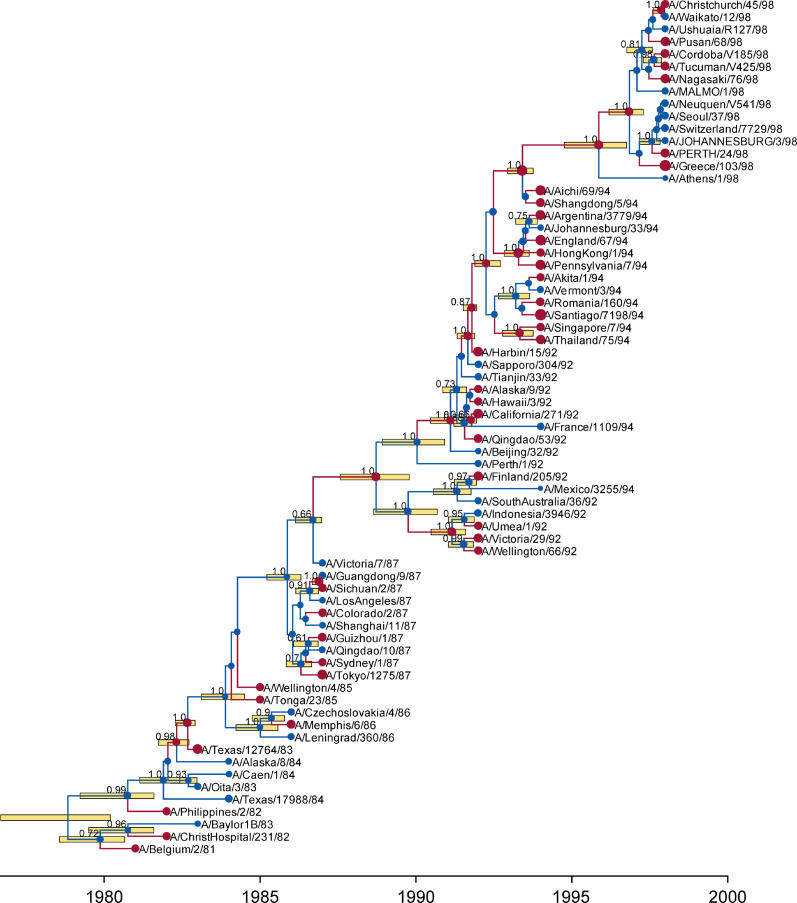
A Tree of 69 Influenza A Virus Sequences Drawn Randomly from the Posterior Distribution The divergence times correspond to the mean posterior estimate of their age in years. The yellow bars represent the 95% HPD interval for the divergence time estimates. Both the mean and 95% HPD of the divergence times were calculated conditional on the existence of the clade defined by the divergence. Each node in the tree that has a posterior probability greater than 0.5 is labeled with its posterior probability. The sampling times of the tips were assumed to be known exactly. Branches colored in red had a posterior rate greater than the average rate, whereas branches colored in blue had a lower-than-average rate.

The estimated coefficient of variation, σ *_r_*, was 0.39 for the dengue virus dataset under the UCLN model. This compares with 0.51 for the influenza A dataset, suggesting that the dengue virus sequences are evolving in a more clocklike manner than the influenza virus sequences. Under the UCED model, both datasets produce an estimated σ *_r_* of 0.99. Under the exponential distribution σ *_r_* should be equal to 1.0 by definition, and the small discrepancy arises because of the discretization procedure described in Materials and Methods. In both datasets the estimated average rate was higher under the UCED model, especially in the internal branches. This elevated rate also corresponded with a lower estimate of the effective population size. Figure 2 shows a tree topology sampled from the posterior of the UCLN analysis of the influenza A virus dataset.

### Marsupials

In addition to the viral sequences, we analyzed a marsupial dataset. The alignment contained concatenated nuclear protein–coding genes ( *APOB, RAG1, IRBP, vWF,* and *BRCA1;* 3,772 bp) for 17 marsupials and seven (outgroup) placental mammals, obtained from Amrine-Madsen et al. [ 31].

The extensions to BEAST for inferring divergence times, described here, are well suited to the marsupial dataset. It possesses some phylogenetic uncertainty, so it is more reasonable to integrate over the posterior distribution of topologies than to assume a single true topology. Furthermore, the dataset includes taxa that have evolved to substantially different sizes, life histories, and niches, which are all hypothesized predictors of molecular rate variation [ 32, 33].

The early fossil record of marsupials [ 34] is poorly known. As a result, point calibrations that utilize the oldest fossils that mark divergences of one group from another are likely to be substantial underestimates, whereas simply defining wide calibration bounds can poorly represent our understanding of the fossil record. We selected prior probability distributions as calibration priors ( Table 3) with the intention of providing realistic assessments of the uncertainty associated with the fossil record [ 35].

**Table 3:**
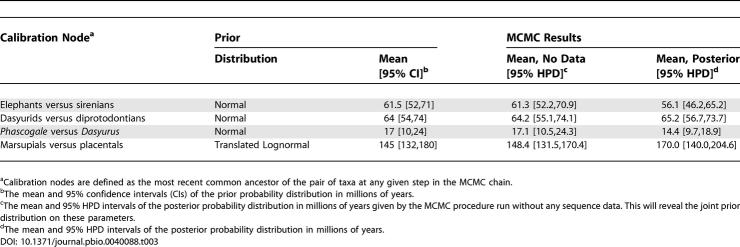
Prior Probability Distributions and Posterior Probability Densities of the Marsupial Calibrations

First, to ascertain the joint prior distribution on the nodes of interest, the four calibration points, the Yule prior, and the reciprocal monophyly constraints were analyzed without any sequence data. The combined results of two runs of 10,000,000 steps are given in Table 3.

In order to analyze the marsupial data, we assumed a general time-reversible [ 36] model of nucleotide substitution with gamma-distributed rate heterogeneity among sites [ 28] and a proportion of invariant sites. In addition, we assumed a UCLN model of rate variation among branches in the tree. A Yule prior on branching rates was employed and the reciprocal monophyly of the ingroup and outgroup was assumed a priori. Four independent MCMC analyses were each run for 10,000,000 steps, resulting in acceptable mixing as determined by Tracer 1.2 [ 23]. These four runs were combined to obtain an estimate of the posterior distribution ( Table 3). The 95% credible set of the marsupial analysis included 12 unique tree topologies, and the maximum a posteriori (MAP) tree topology accounted for 0.32 of the total posterior probability. The estimated rate of the fastest branch in the MAP topology was 2.7 times faster than that of the slowest branch. The mean rate of evolution was 0.944 substitutions per site per billion years (95% HPD: 0.817–1.073). The birth rate of the Yule prior was estimated to be 0.0133 (95% HPD: 0.0035–0.0234).

**Figure 3:**
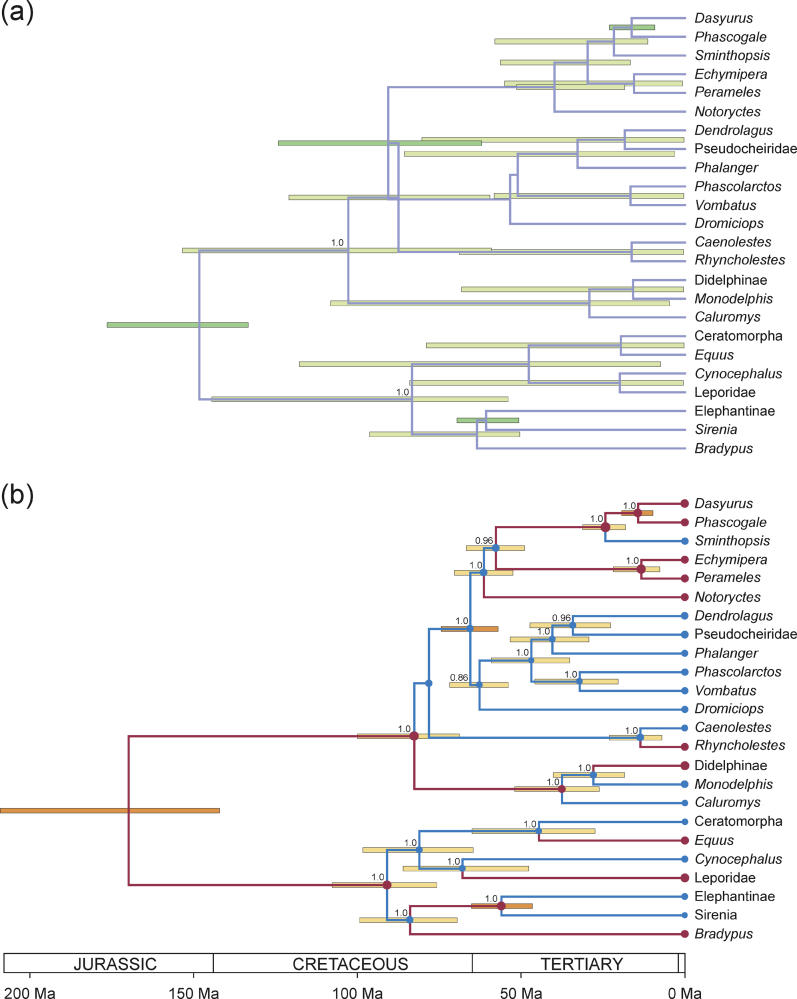
The Analysis of 17 Marsupials and Seven Placental Mammals (A) The combined prior distribution of divergence times for the MAP tree topology. The green bars represent the 95% HPD interval for the divergence times. (B) The posterior distribution of the divergence times. The divergence times correspond to the mean posterior estimate of their age in millions of years. The yellow bars represent the 95% HPD interval for the divergence time estimates. Both the mean and 95% HPD of the divergence times were calculated conditional on the existence of the clade defined by the divergence. Each node in the tree is labeled with its posterior probability if it is greater than 0.5. The three nodes with normally distributed calibration priors are indicated by orange bars. Branches colored in red had a posterior rate greater than the average rate, whereas branches colored in blue had a lower-than-average rate.

There was a slight tendency toward a positive correlation in the rate of parent and child branches but this was not significant (zero was included in the 95% HPD). The coefficient of variation was estimated to be 0.32 (95% HPD: 0.23–0.43), suggesting that the marsupial dataset is more clocklike than both of the virus datasets. Figure 3 shows the (A) prior and (B) posterior distributions of the clades present in the MAP tree topology.

### Autocorrelation of Rates amongst Lineages

We see no autocorrelation for the viruses we analyzed (the HPD interval of the covariance of parent and child branches was [−0.17,0.15] and [−0.18,0.15] for influenza and dengue-4 datasets respectively under the lognormally distributed model of rate variation and [−0.2,0.13] and [−0.19,0.13] for the exponentially distributed model of rate variation). For the marsupial dataset there is a small degree of autocorrelation suggested by the mean estimate, but it is not significantly different from zero (mean: 0.07, HPD: [−0.256, 0.4]). We would expect that larger datasets, particularly of diverse organisms that vary considerably in life-history traits or proofreading mechanisms, might exhibit substantial autocorrelation.

### Assessing Accuracy and Precision with Five Large Datasets

Five large datasets were obtained from previous studies: (1) amino acid alignments of 102 genes from eight bacterial species; (2) nucleotide alignments of 106 genes from eight yeast species [ 37]; (3) nucleotide alignments of 61 genes from nine plants; (4) amino acid alignments of 99 genes from nine metazoans; and (5) 500 nucleotide alignments of non-coding sequence from nine primates. The bacterial dataset was a subset of a larger dataset comprising 730 genes representing 45 species of bacteria [ 38]. Eight species of Proteobacteria were selected due to their close phylogenetic relationship, as well as representation among the 730 genes. A total of 102 genes spanned all eight of the species that were retained for analysis. The plant dataset was taken from a larger dataset comprising 61 genes from 12 taxa [ 39]. Nine species were selected in accordance with the stipulation that their phylogeny was known with almost complete certainty. The metazoan alignment was a subset of a larger dataset comprising 123 genes from 36 eukaryotes [ 40]. Nine metazoan taxa were selected from this dataset so that the tree relating the selected taxa was not in dispute. Genes that were unavailable for one or more of the nine selected taxa were removed, leaving 99 genes in the final dataset used for phylogenetic analysis. The primate dataset was a subset of a 2,160,276 bp alignment of non-coding DNA from 19 mammals [ 41, 42]. The non-primates were removed from the alignment, and sites with a gap in any sequence were removed. The remaining alignment was broken up into 500 alignments of equal length (632 bp). These individual alignments were each intended to represent the data produced by an ordinary phylogenetic study in which a gene fragment has been sequenced from a number of organisms. The question being asked is if we only have one such alignment, how well are we able to reconstruct the phylogenetic relationships of the organisms?

**Figure 4:**
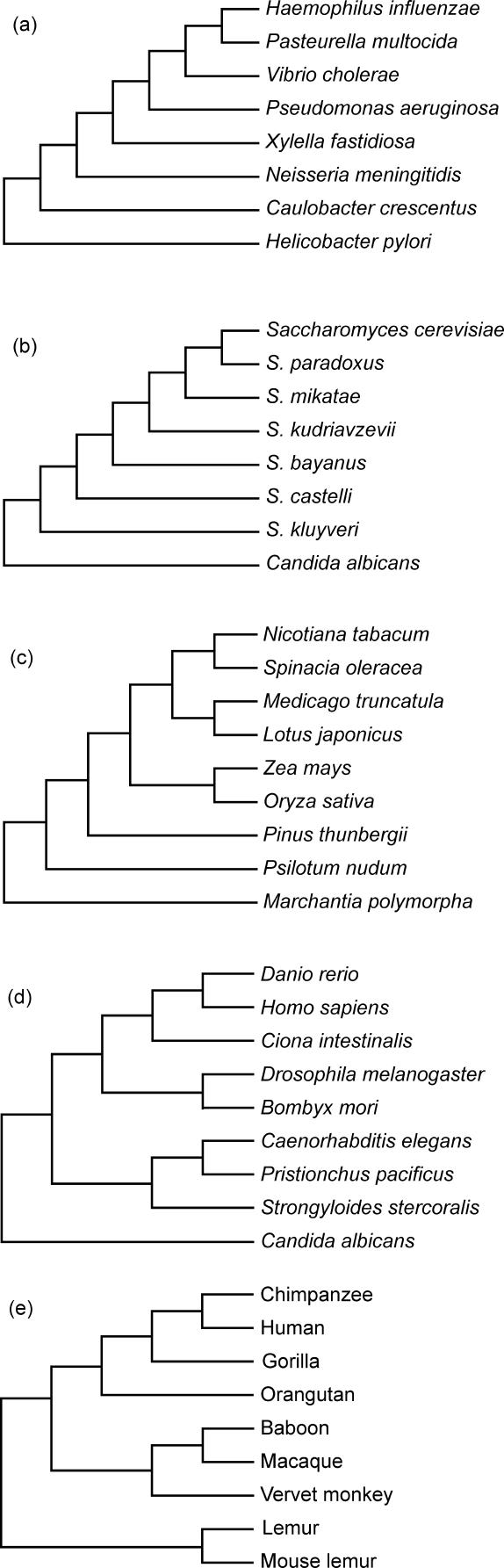
The “True” Phylogenies for the Large Datasets The datasets are as follows: (A) bacterial, (B) yeast, (C) plant, (D) metazoan, and (E) primate.

To assess the accuracy of the phylogenetic methods being tested, estimates of the phylogeny need to be tested against the true phylogeny for each dataset. In order to obtain the best possible estimates of the phylogeny for each dataset, the alignments in each of the five datasets were concatenated. The five concatenated alignments were analyzed under the HKY model of nucleotide substitution with gamma-distributed rate variation among sites and a proportion of invariant sites. Each analysis was run for 5,000,000 MCMC steps, with a discarded burn-in of 500,000 steps. Identical trees were obtained using BEAST with a UCLN model and with MrBayes ( Figure 4). The trees inferred from the plant, metazoan, and primate datasets agree well with the established trees for these groups. However, the bacterial and yeast phylogenies are relatively uncertain [ 37, 43], and the trees inferred from the concatenated alignments are probably the best estimates currently available. Even if these trees turn out to be different from the true evolutionary histories of the studied organisms, we can at least assume that the trees used in this analysis are very near in tree space to the truth, and therefore we would expect our results to be little affected. The yeast tree inferred in this study from concatenated data agreed with that published by Rokas et al. [ 37], also confirmed by Phillips et al. [ 43] under different phylogenetic models.

For each of the five groups of data, each alignment was analyzed using MrBayes (unrooted Felsenstein [UF] model), BEAST with a molecular clock (CLOC); and uncorrelated lognormal relaxed clock (UCLN). The HKY model of nucleotide substitution was assumed, with gamma-distributed rate variation among sites and a proportion of invariant sites. Most analyses were run for 500,000 MCMC steps with 50,000 burn-in steps, although some datasets required 1,000,000 steps with 100,000 burn-in steps. All analyses were checked for convergence using the program Tracer 1.2 [ 23]. The 95% credible set of trees was obtained for each alignment and compared with the “true” trees obtained using the method described above. The accuracy of the methods was considered to be the frequency with which the true tree was contained in the 95% credible set, whereas the average size of the credible sets was taken to represent precision. These terms have statistical definitions, but we take liberties here to facilitate easier interpretation.

All three methods performed poorly in analyses of the bacterial and metazoan datasets. This result is not surprising, however, considering the substantial time depth of these trees. The uncorrelated relaxed-clock method produced the most accurate estimates of phylogeny overall ( Table 4). It outperformed other methods in analyses of the bacterial, yeast, metazoan, and primate data, but the molecular clock method was the most accurate in the analysis of the plant data. A large proportion (76%) of the yeast alignments were significantly non-clocklike (as measured by a likelihood ratio test [ 10] on the true tree topology), which explains the considerable difference in accuracy between the uncorrelated relaxed-clock method (85.8% of the credible sets contain the true tree) and the molecular clock method (67.9% of the credible sets contain the true tree). The superior performance of the molecular clock in the analysis of the plant data, for which the molecular clock was rejected for 67% of the alignments, may be due to the sensitivity of the likelihood ratio test to even small departures from the clock assumption.

**Table 4:**
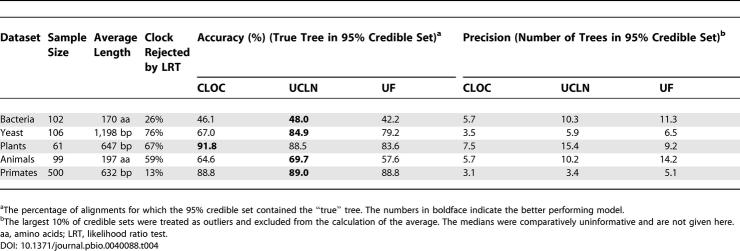
Accuracy and Precision of Phylogenetic Inference Using Three Bayesian Methods: CLOC, UCLN, and UF

In the case of the primate data, all three methods were similarly accurate in estimating phylogenies. This is probably because the data were relatively clocklike, with the molecular clock assumption rejected for less than a third of the alignments. For all of the datasets that were analyzed, the phylogenetic estimates made using a strict molecular clock were the most precise. As expected, the average size of the 95% credible set of trees was always the smallest for the molecular clock method, and nearly always greatest for the unrooted method. Under conditions in which the data more or less conform to a molecular clock, such as the primate data examined in this study, the molecular clock method should be used due to its superior precision.

## Discussion

The relaxed phylogenetics methods described here co-estimate phylogeny and divergence times under a relaxed molecular clock model, thus providing an integrated framework for biologists interested in reconstructing ancestral divergence dates and phylogenetic relationships. The method presented here naturally incorporates the time-dependent nature of the evolutionary process without assuming a strict molecular clock. One of the byproducts of estimating a phylogeny using a relaxed clock is an estimate of the position of the root of the tree, even in the absence of a non-reversible model of substitution [ 44, 45] or a known outgroup.

Recently, a number of authors have begun to investigate the impact of various forms of model misspecification on the accuracy of posterior probabilities of clade support [ 46– 48]. In a Bayesian framework, the absence of a molecular clock assumption (either strict or relaxed) represents a prior belief that the tree topology provides no information about relative branch lengths. We suggest that this represents a poor prior belief, and that Bayesian estimation of phylogeny from short sequences may be biased when the time-dependency of the evolutionary process is not modeled. We would argue that the complex time-dependency of the evolutionary process should not be ignored a priori as has been common practice, but should instead be carefully modeled. This paper represents a first attempt at incorporating a relaxed-clock model into a Bayesian method of phylogenetic inference.

We have presented a large analysis of 102 bacterial, 106 yeast, 61 plant, 99 metazoan, and 500 primate alignments that overall suggests the relaxed-clock models are both more accurate and more precise at estimating phylogenetic relationships than current unrooted methods implemented in MrBayes and other programs. Overall, these initial results suggest that a relaxed phylogenetic approach may be the most appropriate even when phylogenetic relationships are of primary concern and the rooting and dating of the tree are of less interest.

## Materials and Methods

The molecular clock assumption can be relaxed in a variety of ways [ 13– 15, 17, 49– 52]. In Bayesian treatments of the relaxed clock, there is a vector of rates *R*={ *r*_1_, *r*_2_,…, *r*_2 *n*−1 _} and a corresponding vector of node heights **t**={ *t*_1_, *t*_2_,…, *t*_2 *n*−1 _} in units of time. The node height vector, in conjunction with an edge graph, *E,* define an ancestral tree g = { *E*, **t**} in units of time. To convert this tree from units of time to molecular evolutionary units, the rates are either assigned to branches [ 15, 17] or to nodes [ 53, 54]. In both types of models, the prior probability of the rates *f _R_*( *R*| *g*) can be calculated by the product of the probability of each rate *r*_2_ in the tree given the ancestral rate *r _A_*_( *i*)_ and the time Δ *t _i_* between the ancestral and derived rate:



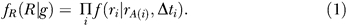



The first such model to be described [ 15] assigned rates to the midpoints of branches and the assumed lognormal prior distribution relating the midpoint of the ancestral branch to the midpoint of the derived branch. Another interesting model is the exponential distribution model of Aris-Brosou and Yang [ 17], which employed an exponential prior distribution on rate *r* with a mean (and therefore standard deviation) equal to the ancestral rate *r _A_,* and with no dependence on the time between the two rates. This second model represents a more punctuated view of change in evolutionary rate, so that only the number of branching events, and not the length of time between events, determines the amount of change in evolutionary rate. In all autocorrelated relaxed-clock models, an additional assumption must be made about the rate at the root. For models that assign rates to nodes, it is necessary to treat the root node in a special way, as it does not have a parent node [ 15]. For models that assign rates to branches, a branch above the root is implied and must be assigned a rate.

In the autocorrelated relaxed-clock models that have been described, including the commonly used lognormal model [ 15, 17, 55], it is also necessary to specify the degree of autocorrelation as a prior. Other prior models of rate change, such as the gamma distribution model and the Ornstein-Uhlenbeck process [ 55], require more than one hyperparameter to be specified, so that selecting suitable values for a particular dataset may be an even more difficult exercise. The effects of varying these hyperparameters are poorly understood [ 22], but there is likely to be a considerable impact on posterior estimates of rates.

### Uncorrelated relaxed clocks

We present an alternative to the autocorrelated prior in which there is, a priori, no correlation of the rates on adjacent branches of the tree. Instead we propose a model in which the rate on each branch of the tree is drawn independently and identically from an underlying rate distribution. We investigate two candidates for the rate distribution among branches:



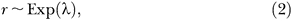





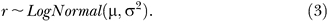



These uncorrelated priors can be framed in a hierarchical Bayesian framework, as with the autocorrelated priors. In this scenario the exponential version of uncorrelated relaxed clock would have a prior probability on the rate vector of:



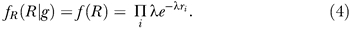



This model corresponds to an exponential prior distribution on rate *r _i_* with a mean (and therefore standard deviation) equal to λ ^−1^ and no dependence on either the rate of the previous branch or the time between the two rates. The parameter λ is a hyperparameter that is fixed and not estimated via MCMC, and represents a prior statement about both the mean and the variance of branch rates. This prior reflects a punctuated view of change in evolutionary rate, so that the prior expectation of the rate at all branches is the same, with no autocorrelation between adjacent branches. Notice that the posterior distribution of rates among branches need not be the same as the prior in this setup and that autocorrelation may exist in the posterior, even though it is not specified in the prior.

Instead of framing Equations 2 and 3 as prior distributions in a hierarchical Bayesian framework, they can instead be reformulated as a full likelihood model. In this case, the branch rates are not independent random variables with a prior distribution, but are instead constrained so as to fit one of the distributions in Equations 2 and 3 exactly. The parameters of the rate distribution are no longer hyperparameters of a prior distribution, but are instead parameters of the likelihood model. This is closely analogous to the common way in which rate heterogeneity among sites is treated [ 28].

### Priors on phylogeny

A particular requirement of Bayesian phylogenetic inference is the responsibility given to users to specify a prior probability distribution on the shape of the phylogeny (node ages and branching order). This can be either a benefit or a burden, largely depending on whether an obvious prior distribution presents itself for the data at hand. For example, the coalescent prior [ 56, 57] is a commonly used prior for population-level data and has been extended to include various forms of demographic functions [ 58, 59], sub-divided populations [ 60], and other complexities. Traditional speciation models such as the Yule process [ 61] and various birth–death models [ 62, 63] can also provide useful priors for species-level data. Such models generally have a number of hyperparameters (for example, effective population size, growth rate, or speciation and extinction rates), which, under a Bayesian framework, can be sampled to provide a posterior distribution of these potentially interesting biological quantities.

In some cases, the choice of prior on the phylogenetic tree can exert a strong influence on inferences made from a given dataset [ 64]. The sensitivity of inference results to the prior chosen will be largely dependent on the data analyzed and few general recommendations can be made. It is, however, good practice to perform the MCMC analysis without any data in order to sample wholly from the prior distribution. This distribution can be compared to the posterior distribution for parameters of interest in order to examine the relative influence of the data and the prior ( Figure 3).

### Bayesian inference

The full Bayesian sequence analysis with an uncorrelated relaxed-clock model allows the co-estimation of substitution parameters, relaxed-clock parameters, and the ancestral phylogeny. The posterior distribution is of the following form:



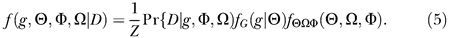



The vector Φ contains the parameters of the relaxed-clock model (e.g., μ and σ ^2^ in the case of lognormally distributed rates among branches). The term Pr *D*|g,Φ,Ω is the standard Felsenstein likelihood, where *g* is a tree with branch length measured in units of time. For the purposes of calculating this likelihood, branch lengths are converted to units of substitutions by multiplying the rates defined by Φ with the internode distance between node *i* and parent node *j* in tree *g*. The tree prior, *f _G_*( *g*|Θ), can either be a coalescent-based prior [ 30, 65] for within-population data or some other appropriate prior if the sequences come from multiple populations/species [ 55]. The vector Θ contains the hyperparameters of the tree prior. The vector Ω contains the parameters of the substitution model (such as transition/transversion ratio, κ; shape parameter for gamma-distributed rates among sites, α; and proportion of invariant sites, *p _inv_*).

We summarize the posterior density in Equation 5 using samples ( *g*,Θ,Φ,Ω) ∼ *f* obtained via MCMC. If, for example, the divergence times are of primary interest then the other sampled parameters can be thought of as nuisance parameters, and vice versa.

The formulation in Equation 5 implies that the branch-rates could be integrated analytically in the Felsenstein likelihood. Although this could be accomplished relatively easily by discretizing the rate distribution and averaging the likelihood over the rate categories on each branch, we elected to do the integration using MCMC. This was achieved by assigning a unique rate category *c* ∈ 1,2,…,2 *n*−2 to each branch *j* of the tree. During the calculation of the likelihood the rate category *c* is converted to a rate by the following method:



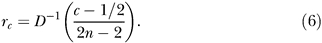



**Figure 5:**
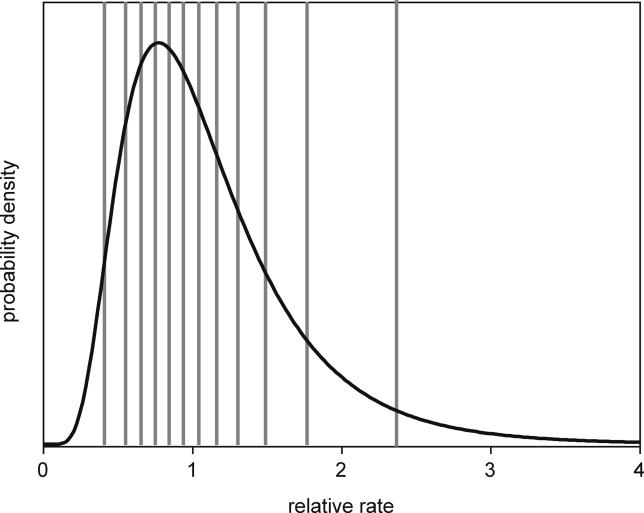
A Lognormal Distribution Discretized into 12 Rate Categories Each of the 12 categories has equal probability ( *p* = 1/12). The *i* ^th^ rate category (numbered from left to right) corresponds to the (I − 0.5)/12 quantile of the lognormal distribution.

The function *D* ^−1^( *x*) is the inverse function of the probability distribution function, *D*( *x*)= *P*( *X*≤ *x*), of the relaxed-clock model specified by Equations 2 and 3. This discretization of the underlying rate distribution is illustrated in Figure 5 for a lognormal distribution with 12 rate categories (sufficient for a tree of seven tips). To integrate the branch rates out, the assignment of rate categories *c* to branches was sampled via MCMC.

### Model selection

One issue that remains largely unresolved in this piece of work is the issue of model comparison and model selection. Within a Bayesian framework, Bayes factors are usually regarded as the correct way to deal with model selection. Typically this involves a technique known as reversible-jump MCMC. We have not implemented this, but we do plan on developing a reversible-jump MCMC version of this framework in the future. Typically model selection is easy when one model produces a much better fit. Because all of the models for rate variation examined here differ by one free parameter at most, a simple comparison of the average log posterior probabilities will usually be revealing. It is only when the log posteriors are very similar and the results are qualitatively different between the two models that model selection becomes an issue. This combination of conditions did not occur in any of our real datasets.

### Proposing new states in the MCMC kernel

The MCMC must sample the tree topology, the divergence times, and the individual parameters of the substitution model and tree prior(s). Therefore, a series of proposal distributions (often called “moves”) needs to be employed. Our MCMC implementation employs an array of moves, each of which is designed to explore a certain subspace in the overall parameter/model space being explored. For example, some moves propose local changes to the tree topology while keeping the coalescent interval and all the other parameters constant. Some moves propose a change to a single substitution parameter (such as the shape parameter of the gamma distribution) while keeping everything else constant. The general scheme is to (1) choose a random move with a probability proportional to a specified weight, then (2) apply the move to the current state, and (3) assess the relative score of the new state. The new state is adopted if it has a higher posterior probability; otherwise it is adopted with probability equal to the ratio of its posterior probability to the posterior probability of the previous state. The weights allow the researcher to favor certain moves which can help with the performance of the MCMC, but generally the default weights give good results. Most of the moves used in our MCMC implementation have been previously described [ 30]. The two new moves involve sampling the rate categories of the branches (a random pair of branches are chosen and their categories are swapped) and dealing with rate categories of branches when a change to the tree topology is made. (We implement two alternatives: keeping all the rate categories the same when a subtree is moved or performing a single rate swap simultaneously with a tree topology change.) These moves are very simplistic, and we suspect that better proposal distributions exist. We have found a small number of datasets in which our current proposal distribution does not work well. Nevertheless, for a large number of datasets including the ones presented in this paper, our scheme performs more than adequately as assessed by repeated runs and estimation of integrated autocorrelation times.

### Summarizing the posterior distribution

The output of an MCMC analysis is a set of samples from the posterior distribution. In the case of the uncorrelated relaxed-clock models described above, the posterior distribution is a distribution over tree topologies, dates of divergence, branch rates, and parameters of the rate and substitution models. This complex set of samples can be summarized in many ways. One of the simplest summaries of the branch rate distribution is to sample the coefficient of variation (σ *_r_*; the standard deviation divided by the mean) of the branch rates. Under the exponential model, σ *_r_ = 1* by definition; under the lognormal model, σ *_r_* gives a measure of the degree of clocklikeness of the data. If σ *_r_* = 0 then the data are perfectly clocklike, whereas larger values correspond to increasing rate heterogeneity among branches. A posterior estimate of σ *_r_* can be easily calculated:



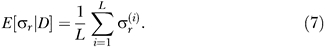



This is the simple average of the calculated 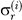 over all *L* samples in the estimated posterior distribution. In addition, 95% HPD limits can also be calculated. In a similar manner, marginal posterior estimates can be calculated for



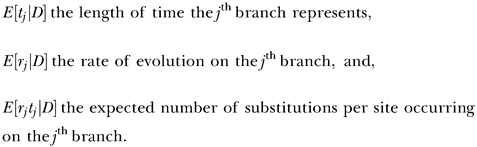



Some subtlety in the interpretation of the posterior distribution of rates is required because both the amount of time a branch represents, *t _j_,* and the rate of evolution along the branch, *r _j_*, are random variables in the MCMC analysis. For example, in general, *E*[ *r _j_t _j_*| *D*] ≠ *E*[ *r _j_*| *D*]· *E*[ *t _j_*| *D*]. For the purposes of this paper, when we refer to the average rate for a set of branches *B* (such as the set of external branches or the set of internal branches), we define it as the weighted average:



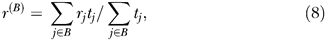



rather than the simple unweighted average 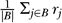 . Thus the posterior estimate of average rate over the whole tree is *E*[ *r* ^( *T*) ^| *D*], where *T* = {1,2,…,2 *n*−2}. In general, this will be different from the mean of the underlying rate distribution because the rate at each branch is weighted by the time represented by the branch. The justification for this is that the overall rate is best summarized by the total amount of substitutions over the total amount of time, which is what Equation 8 calculates.

### Calibrating the rate of evolution

In the above discussion on rate models, it was assumed that it is possible to estimate absolute rates of evolution and the variance in absolute rates. In fact, even under a molecular clock assumption, the divergence times and the overall substitution rate can only be separately estimated if there is a source of external calibration information. In the framework described here, this information can come from one of three sources: (1) Prior information on the age of internal nodes: In a phylogenetic context, calibration information is often obtained by assigning the age of a known fossil to a particular internal node [ 2]. Uncertainty in the association between an internal node and the fossil record can be accommodated by providing a prior probability distribution for the age of the node. Previous studies have used a uniform distribution with upper and lower bounds on the age [ 54], although other distributions may be suitable [ 35]. In the above Results section, we presented examples in which calibration times are treated with parametric prior distributions (normal and lognormal). Assigning an age to a particular node is only possible when the tree itself is assumed to be known and fixed, a limitation of previous relaxed-clock implementations [ 15, 17, 54]. In the framework presented here, the tree itself is being sampled and thus we cannot define the age of a particular internal node. Instead we specify the age, or the prior distribution of age, for the most recent common ancestor of a set of taxa. Every time a new tree is proposed in the MCMC chain, the most recent common ancestor of the specified taxa is located in the tree, and the prior probability of the age of this node is used to assess the acceptance probability of the proposed tree. (2) Known ages of the sequences: Recently it has also been demonstrated that calibrations can be associated with the sequences at the tips of the tree if they are sampled at significantly different times [ 29, 30, 66] with respect to their rate of evolution. Again, there may be uncertainty in calibration dates [ 67]. The RNA virus data in this study provide examples of this form of calibration information. (3) A strong prior on the substitution rate: If the mean substitution rate is known from a previous study on independent data, then this can be incorporated as prior knowledge. In the simplest case this can be achieved by fixing the rate of evolution to a known value. It is also straightforward to sample the rate from a parametric distribution obtained from a previous (independent) analysis [ 68, 69]. If there is no prior information about the mean substitution rate, then it can be fixed to 1, resulting in time being in units of substitutions per site.

All of these forms of calibration information can be incorporated into our MCMC implementation either on their own or in any combination, as appropriate.

## Supporting Information

Protocol S1 Relaxed Phylogenetics and Dating with Confidence (167 KB DOC).

## Abbreviations

ACED: autocorrelated exponential distribution
ACLN: autocorrelated lognormal distribution
bp: base pairs
CLOC: strict molecular clock
HPD: highest posterior density
MAP: maximum a posteriori
MCMC: Markov chain Monte Carlo
UCED: uncorrelated exponential distribution
UCLN: uncorrelated lognormal distribution
UF: unrooted Felsenstein
