# Supplementary material for: Relaxed Phylogenetics and Dating with Confidence
Source: PLoS Biol. 2006 Mar 14;4(5):e88. doi: 10.1371/journal.pbio.0040088 (PMC1395354; doi:10.1371/journal.pbio.0040088)
Supplement: Supplementary file 1 [file pbio.0040088.sd001.doc]

**Supplementary Information for “Relaxed phylogenetics and dating with confidence”**

Alexei J. Drummond*, Simon Y. W. Ho, Matthew J. Phillips, and Andrew Rambaut

Department of Zoology, University of Oxford,

South Parks Road, Oxford, OX1 3PS, UK.

[alexei.drummond@zoo.ox.ac.uk](mailto:alexei.drummond@zoo.ox.ac.uk)

[simon.ho@zoo.ox.ac.uk](mailto:simon.ho@zoo.ox.ac.uk)

[matthew.phillips@zoo.ox.ac.uk](mailto:matthew.phillips@zoo.ox.ac.uk)

[andrew.rambaut@zoo.ox.ac.uk](mailto:andrew.rambaut@zoo.ox.ac.uk)

Group web site: http://evolve.zoo.ox.ac.uk/

BEAST web site: http://evolve.zoo.ox.ac.uk/beast/

**Dengue virus data**

69 E gene sequences; Dengue virus serotype 4, from Puerto Rico; see the following reference for details:

Bennett SN, Holmes EC, Chirivella M, Rodriguez DM, Beltran M, et al. (2003) Selection-driven evolution of emergent dengue virus. *Mol Biol Evol* **20**: 1650-1658

The alignment used is available as supplementary information with the name: “DENV4.E.PRico.nex”

**Influenza A virus data**

69 hemagglutinin (HA) sequences; collected from http://www.flu.lanl.gov/

The alignment used is available as supplementary information with the name: “IVA.HA.Human.81-98.nex”

The strains used were:

A/Belgium/2/81

A/ChristHospital/231/82

A/Philippines/2/82

A/Baylor1B/83

A/Oita/3/83

A/Texas/12764/83

A/Alaska/8/84

A/Caen/1/84

A/Texas/17988/84

A/Tonga/23/85

A/Wellington/4/85

A/Czechoslovakia/4/86

A/Leningrad/360/86

A/Memphis/6/86

A/Colorado/2/87

A/Guangdong/9/87

A/Guizhou/1/87

A/LosAngeles/87

A/Qingdao/10/87

A/Sichuan/2/87

A/Shanghai/11/87

A/Sydney/1/87

A/Tokyo/1275/87

A/Victoria/7/87

A/Alaska/9/92

A/Beijing/32/92

A/California/271/92

A/Finland/205/92

A/Harbin/15/92

A/Hawaii/3/92

A/Indonesia/3946/92

A/Perth/1/92

A/Qingdao/53/92

A/Sapporo/304/92

A/SouthAustralia/36/92

A/Tianjin/33/92

A/Umea/1/92

A/Victoria/29/92

A/Wellington/66/92

A/Aichi/69/94

A/Akita/1/94

A/Argentina/3779/94

A/England/67/94

A/France/1109/94

A/HongKong/1/94

A/Johannesburg/33/94

A/Mexico/3255/94

A/Pennsylvania/7/94

A/Romania/160/94

A/Santiago/7198/94

A/Shangdong/5/94

A/Singapore/7/94

A/Thailand/75/94

A/Vermont/3/94

A/Athens/1/98

A/Christchurch/45/98

A/Cordoba/V185/98

A/Greece/103/98

A/JOHANNESBURG/3/98

A/MALMO/1/98

A/Nagasaki/76/98

A/Neuquen/V541/98

A/PERTH/24/98

A/Pusan/68/98

A/Seoul/37/98

A/Switzerland/7729/98

A/Tucuman/V425/98

A/Ushuaia/R127/98

A/Waikato/12/98

**Marsupial data**

Concatenated nuclear protein-coding genes (*APOB*, *RAG1*, *IRBP*, *vWF*, *BRCA1*; 3,772 bp) for 17 marsupials and seven (outgroup) placental mammals, obtained from:

Amrine-Madsen H, Scally M, Westerman M, Stanhope MJ, Krajewski C, et al. (2003) Nuclear gene sequences provide evidence for the monophyly of australidelphian marsupials. *Mol Phylogenet Evol* **28**: 186-196.

GenBank accession numbers. Note that 11 taxa are chimeric, utilizing sequences from related species/genera.

| Taxon | Species | Common name | APOB | RAG1 | IRBP | vWF | BRCA1 |
| --- | --- | --- | --- | --- | --- | --- | --- |
| Didelphinae | Didelphis virginiana | Virginia opossum | AF548432 |  | Z11814 | AF226848 | AF497261 |
|  | Lutreolina crassicaudata | Thick-tailed opossum |  | AY243390 |  |  |  |
| Monodelphis | Monodelphis domestica | Gray short-tailed opossum | AY243431 | U51897 | AF257694 | AY243415 | AY243453 |
| Caluromys | Caluromys philander | Woolly opossum | AY243419 | AY243385 |  | AY243404 | AY243444 |
|  | Caluromys lanatus | Western woolly opossum |  |  | AF257690 |  |  |
| Caenolestes | Caenolestes fuliginosus | Silky shrew opossum | AY243418 | AY243384 | AF025381 | AY243403 | AF355794 |
| Rhyncholestes | Rhyncholestes raphanurus | Chilean shre opossum | AY243432 | AY243399 | AY24340 | AY243416 | AY243454 |
| Dromiciops | Dromiciops gliroides | Monito del monte | AY243423 | AY243389 | AF025384 | AY243407 | AY243446 |
| Sminthopsinae | *Sminthopsis sp.* | Dunnart | AY243428 |  |  | AY243413 |  |
|  | *Planigale sp.* | Pygmy marsupial mouse |  | AY243396 | AY243438 |  | AY243451 |
| *Dasyurus* | *Dasyurus albopunctatus* | New Guinean quoll | AY243430 | AY243398 |  | AY243414 | AY243452 |
|  | *Dasyurus maculatus* | Spotted-tailed quoll |  |  | AY243439 |  |  |
| *Phascogale* | *Phascogale tapoatafa* | Brush-tailed phascogale | AY243427 | AY243395 | AF025382 | AY243412 | AF355795 |
| *Notoryctes* | *Notoryctes typhlops* | Marsupial mole | AY243424 | AY243391 | AF025385 | AY243408 | AY243447 |
| Echymipera | Echymipera kalubu | Spiny bandicoot | AY243420 | AY243386 | AF025383 | AY243405 | AF355796 |
| *Perameles* | *Perameles nasuta* | Long-nosed bandicoot | AY243426 | AY243394 | AY243437 | AY243411 | AY243450 |
| *Vombatus* | *Vombatus ursinus* | Common wombat | AY243429 | AY243397 | AF025386 | AF497260 | AF284031 |
| *Phascolarctos* | *Phascolarctos cinereus* | Koala | AY243421 | AY243387 | AY243434 | AY243406 | AY243445 |
| *Phalanger* | *Phalanger orientalis* | Gray cuscus | AF548431 | AY243393 | AY243436 | AY243410 | AY243449 |
| Pseudocheiridae | *Pseudochirops cupreus* | Coppery ringtail possum |  | AY243392 | AF025387 | AY243409 | AY025448 |
|  | *Pseudochirops archeri* | Green ringtail possum | AY243425 |  |  |  |  |
|  | *Pseudocheirus herbertensis* | Herbert river ringtail |  |  |  |  | AY025448 |
| Macropodinae | *Dendrolagus goodfellowi* | Goodfellow’s tree kangaroo | AY243422 |  | AY243435 |  |  |
|  | *Dendrolagus Inustus* | Grizzled tree kangaroo |  | AY243388 |  |  |  |
|  | *Macropus giganteus* | Eastern grey kangaroo |  |  |  | AJ224670 |  |
|  | *Macropus rufus* | Red kangaroo |  |  |  |  | AF284033 |
| *Cynocephalus* | *Cynocephalus volans* | Philippine flying lemur | AF548401 |  |  |  |  |
|  | *Cynocephalus variegatus* | Malayan flying lemur |  | AY011897 | Z11807 | U31606 | AF019081 |
| Leporidae | *Oryctolagus cuniculus* | Rabbit |  | M77666 |  |  |  |
|  | *Lepus americanus* | Snowshoe hare | AF548415 |  |  |  |  |
|  | Lepus capensis | Brown hare |  |  |  |  | AF284005 |
|  | Lepus crawshayi | Savanna hare |  |  | AJ427250 | AJ224669 |  |
| Ceratomorpha | Ceratotherium simum | White rhinoceros |  | AY011913 |  | U31604 |  |
|  | *Diceros bicornis* | Black rhinoceros | AY243375 |  |  |  | AF284011 |
|  | *Tapirus pinchaque* |  |  |  | AF179294 |  |  |
| Equus | *Equus caballus* | Horse | AF548405 | AF447516 | U48710 | AF034691 | AF284010 |
| Sirenia | *Dugong dugon* | Dugong | AF548410 |  | U48583 | U31608 | AF284019 |
|  | *Trichechus manatus* | Caribbean manatee |  | AY011874 |  |  |  |
| Elephantidae | *Loxodonta africana* | African elephant |  | AY243402 |  |  |  |
|  | *Elephas maximus* | Asian elephant | AF548407 |  | AY243443 | U31611 | AF284022 |
| Bradypus | *Bradypus tridactylus* | Three-toed sloth | AF548427 | AY243401 | U48708 | U31603 | AF284002 |

The alignment used is available as supplementary information with the name: “Marsupials.nex”

*Fossil calibration*

We calibrate rates on the marsupial tree from two internal divergences among marsupials and an external divergence among placental mammals. Instead of point calibrating from the oldest fossils that mark divergences of one group from another (necessarily an underestimate), we provide normally distributed calibration prior distributions. The lower bound of the prior 95% confidence intervals are defined as the earliest appearance of a fossil that can be placed on one of the lineages descended from the node being calibrated. Upper bounds are then determined by how much further back one has to look before quite well sampled fossil assemblages in potential geographic regions of origin contain no putative members of these groups, but contain stem members or ecological equivalents.

1. Dasyurids vs. diprotodontians. This is the basal split among Australasian marsupials. Lower: (54Ma) Dasyuromorphs/peramelemorphs are first recorded among the Murgon fauna [1]. Upper: (74Ma) Putative australidelphians (e.g. Khasia) are known from the early Palaeocene of South America. Extending the range back to the mid-Campanian covers the possibility of australidelphians not appearing in later Cretaceous fossil faunas due to poor sampling.
2. Phascogale vs Dasyurus. This is the split between the dasyurid sub-families Phascogalinae and Dasyurinae. Lower: (10Ma) Van Dyck [2] suggested an Early Miocene dasyurid to have Phascogaline affinities, though this is not well accepted. Instead we refer to the mid-late Miocene dasyurine-like and antechinus-like fossils of Archer [3] as a more conservative lower bound. Upper: (24Ma) No putative dasyurines, phascogalines (or their stem members) are known from latest Oligocene fossil faunas (or before), hence we place the upper bound at the Oligocene/miocene boundary.
3. Elephants vs. sirenians. Lower: (52Ma) Early Eocene stem-sirenian fossils [4] from the Carribean provide a more conservative bound than do the questionably dated late-Palaeocene proboscideans [5]. Upper: (71Ma) Asher *et al.* [6] suggest that the affinities of such afrotherian mammals lie with “condylarths”. If proboscideans and sirenians are independently derived from these, then their origins may be traced back to the late Cretaceous, but not prior to the Maastrichtian.
4. Marsupials vs placentals (the root and the basal therian split): A lognormal prior distribution that is concentrated closer to the start of the known fossil range and has a tail to cover the time up to the less substantive maximum bound provides a closer reflection of our understanding of early therian fossil history than does a normal distribution. A translated lognormal with a mean of 3.0 and standard deviation of 0.5 was used to provide a lower cut-off (125Ma), a mean (145Ma) and an upper bound for the 95% CI (180Ma). The lower cut-off corresponds to the oldest fossils that can be confidently assigned to either the placental or marsupial stem lineages [7]. The mean allows the highest density to be given to when fossils that appear to be very close to expectations for the last common ancestor of the two lineages are found. The upper bound acknowledges that only stem therians are known (or can be inferred) to have been present in faunas pre-dating the Middle Jurassic.

*References*

1. Godthelp H, Archer M, Cifelli R, Hand SJ, Gilkeson CF (1992) Earliest known Australian Tertiary mammal fauna. *Nature* **356**: 514-516.
2. Van Dyck S (1989) Biting remarks on the Riversleigh antechinus. *Riversleigh Notes* **7**: 2-3.
3. Archer M (1982) Review of the dasyurid (Marsupialia) fossil record, integration of data bearing on phylogenetic interpretation and suprageneric classification. In: Archer M, editor. *Carnivorous Marsupials Vol 1*. Sydney: Royal Zoological Society of New South Wales. pp. 397-443.
4. Domning DP (2001) The earliest known quadrupedal sirenian. *Nature* **413**: 625-627.
5. Gheerbrant E, Sudre J, Cappetta H (1996) A Palaeocene proboscidean from Morocco. *Nature* **483**: 68-70.
6. Asher RJ, Novacek MJ, Geisler JH (2003) Relationships of endemic African mammals and their fossil relatives based on morphological and molecular evidence. *Journal of Mammalian Evolution* **10**: 131-194.
7. Luo Z-X, Wible JR, Yuan C (2003) An Early Cretaceous tribosphenic mammal and metatherian evolution. *Science* **302**: 1934-1939.
